# General Anæsthesia Scarcely Justifiable in Extraction of Teeth

**Published:** 1883-09

**Authors:** J. Hardeman

**Affiliations:** Muscatine, Iowa.


ARTICLE II.

### GENERAL ANÆSTHESIA IS SCARCELY JUSTIFIABLE IN EXTRACTION OF TEETH.

BY J. HARDEMAN, D. D. S., MUSCATINE, IOWA.

Upon the first page of your May issue is a very ably written communication by Dr. J. H. Coyle, D. D. S., advocating the use of Anæsthetics in dentistry.

I am sorry I have not the personal acquaintance of the writer, as he manifests in this communication many traits of commendable merit, but my present convictions being so much at variance with the Doctor's views in regard to the justification of the use of Anæsthesia in dental practice, I beg leave, therefore to express some of my exceptions etc., upon this matter.

I think I can appreciate the Doctor's position. Twenty to thirty years ago I was an ardent advocate for the use of

Sul. Ether and Chloroform as he is now for any Anæsthetic agent; and, I used them as frequently and as recklessly as he does these and the other agents he mentions.

The Doctor is more reliant than those usually claiming to be experts in the use of these powerful agents. He says he makes "no distinction whatever as to age, sex or condition"—"lesions of heart or lungs" even being no barrier. He fears neither Chloroform with its long black list, or its more recent ally Bromide of Ethel that marks the largest per centum of fatality of any upon record of this class.

It is always well in the investigation of any question connected with vital organism to not lose sight of physiological, pathological and therapeutical facts. Narcotism, (say from Chloroform,) paralyzes the nerve force of the parts upon which it acts, sensation is lost; motion is lost, and consciousness is more or less suspended. Man possesses voluntary and involuntary nerve power, and it is by this latter (involuntary) that life is continued in an individual. Paralysis of the sympathetic major, stops the beating of the heart, the respiration of the lungs, etc., and thus the seat of vitality is dethroned, life suspended, and death entering at the door. We do not wish to over-draw, but desire to present to mental view the lay of the ground. The fifth pair of nerves is paralyzed; the optic and aurum nerves are paralyzed; even those arising from the medulla spinalis are benumbed. The great toe may even be severed without the least cephalic phenomenon, and I ask what good reason, or can any reason be given, if the nerve centres, and even the spinal cord, are paralyzed, why will, or how can the medulla oblongata (the seat of this involuntary or automatic power) remain active? If a general Anæsthetic suspends the sensation of an organ or a limb, it is evident that the force causing this is, in effect, at the neutral seat or base; and if the lateral and upper parts of the brain become paralyzed, why may not the lower part, where the sympathetic has its seat or origin, and if this lower or basal position of the brain *is paralyzed, then what?*

In autopsy of fatal cases from chloroform (and most other agents of the kind) the brain is invariably found blanched, while the lungs and heart are engorged with blood, plainly showing that the paralyzed heart failed to continue to force the natural sanguinious stimulant to the head. And this is found to be the case whether the individual dies in a recumbent position or any other.

We admit that many pass successfully through anæsthesia and that comparatively few die ; but the wonder is that the reverse is not the result. That the nature of blood vitality can, for a time, sustain this shock, and if again the blood is poured upon the medulla oblongata will revive again that organ to re-action, must be the fact and this is made manifest by chloroforming a rat until breating ceases, when holding him up by the tail he revives, and then laying him down, vitality is suspended as before. But repetition of this Helaton's plan will not always succeed, however good the principle and commendable the practice where danger threatens.

At best, anæsthesia is a step in the dark, and always partakes more or less of pure empiricism. The landmarks of prognosis or of caution are not sufficiently reliable. The Doctor says, "*always stop short of sturterous breathing.*" Who knows when sturter is going to begin? We only recognize it when it *has begun.* A clergyman in Ohio died at the second chloroform inspiration. A strong man died in the hands of two of our best physicians here, while in the horizontal position, after taking but one or two inspirations—no sturter, but simply straightening himself out and ceasing to breathe. Myself was present when a thirteen-year-old boy took several inspirations of a combination of Sul. Ether and Chloroform and suddenly screamed out loudly, "*I am dying!*" and as the last word left his mouth his eyes blanched in death. Six Medical Doctors present and doing their best, but could not call back the vital spark, and this patient was lying down from the start of the administration. At other time death takes place after an apparent revival; a few hours, or the next day has found the patint sinking, and all stimulants or other restoratives proving futile.

I must also diffier with Dr. Coyle in regard to the comparative danger of any agent of general anæsthesia may have upon the patient in regard to structural formation. Organic lesions of the heart especially, must enhance the liability to fatal results in proportion to the degree of abnormability; because patients known to be more or less so afflicted, and pass thus successfully through anæsthesia, do not tell the story always how *very nearly* they then came of passing through "this door standing ajar." It is very self-evident to a reflective mind that a heart with thinned and weakened walls, or one with ossified valves, will not be as fully able to throw off the super-accumulated sanguinious mass caused by the narcotism, as would be the normally complete one. For, no doubt, in each case a degree of suspended power attends the heart's action in anæsthesia, as is manifested, unmistakably by the fall in the tone and frequency of the pulse. Not only is this so from direct narcotism from the drug, but it is enhanced from the *imperfect decarbonization of the blood.*

And just here we would refer to the habit of many, as well as your respected correspodent, of "*crowding*" an agent upon the patient without an admixture of air. "In spite of struggles and resistance" the inhalation is to be crowded upon them. This is recommended as correct for Nitrous Oxide and Eythl Bromide by Dr. Coyle, and by not a few others for any agent used for general anæsthesia. This practice is pernicious, and, to me, bordering too much upon the barbarous. The patient is simply suffocated; thrown into a state of asphyxia rather than that of anæsthesia. I have witnessed some of this kind of treatment, and could readily recognize the state of condition by the purple and livid pallor, and without doubt there was a relative and impending danger. I attended an exhibition by a traveling "laughing gas" peddler at one time, years ago, and by dint of a chance that presented, had a peep into—did see the doings behind the curtain. After several volunteer parties had inhaled from, and exhaled into the bag, the Professor found it becoming too lank, and, in company with his "John," retired to replenish the gas from the "chemicals." When behind the courtain, the Professor applied the bag to his mouth and expired unto it for a number of times and then handed it to "John" and he in turn expired lustily into the bag and returned it to the Professor, who, with a few more puffs started with a round, full bay of "laughing gas." He at once applied it to the mouth of a young doctor, who soon after began to declaim from Shakespeare. Next, a middle-aged lawyer became stupefied with this filthy, effete carbonic acid, and he devoutedly knelt in silence, and upon recovery remaked, "I don't see much *laughing gas* in that."

Carbonic acid, or air sur-charged with it, if inspired will of course suspend amination, and will do it permanently, too, if carried far enough. And this is often the effective agent used by those who make a funnel-shaped face cap with a napkin, with paper between the folds to closely shut out the air while trying to administer on anæsthetic, *and it is enough to make them "struggle."*

The Doctor also indicates that in the selection of the agent, regard should be had rather to the relative duration of effect than that of danger. It is well if a choice can be made from a class of admissible agents, that they be thus selected to suit special requirements, but it strikes me that from the evidence so commonly known, that there is a great difference in the degree of *attending danger*. It is certain that chloroform numbers its victims by the hundreds—a per centum that, if strictly known, would be simply shocking to civilization. A larger per cent. of fatality has been laid to the dental than the medical practice. This, I think, is erroneous and unfair. Every case occurring in dentistry is heralded abroad, and gets into the press, and often into courts, while but a mere fraction from the surgeons ever see the records or a jury—are reported as "dying in the operation," and this is frequently the last of it. Who knows of a fatal case occurring in the dental chair and allowed to pass without the calling of a coroner's jury? And how many ever see a coroner's jury called to set upon a fatal case occurring in the general medical practice? Chloroform has a *black* record if it were all told, and, for its age, Ethyl Bromide is worse. It was a long time after the introduction of Sul. Ether and Chloroform for anæsthesia before a case of fatality occurred. Not so with this Bromide of Ethyl. To be brief, I would place the four principle agents used for degree of safety in the following order: Nitrous Oxide, Sul. Ether, Chloroform, Bromide of Ethyl. The first of these I make frequent use of in my practice, the second occasionally, the third *no more* and the fourth I do not expect ever to use at all.

Pain is often a blessing rather than a curse; and the amount of suffering attending extraction is much less where the custom to use anæsthetics, and other means that tend to educate an intensity of dread is not in vogue, and to have the patient's intelligence and consciousness, and a reasonable allotment of time in an operation, how much better, *and how much more safe.*

Dr. C——, very correctly recommends the incumbent position, and a caution to guard against blood getting into the rima glottidis (he inadvertently said "epiglottis,") but the *greater* danger is during the anæsthetic hurry and confusion, the tooth may fly from the points of the forceps and lodge in the larynx. This misfortune can occur from the most experienced operator; and, of course, under any ordinary conditions, but the liability is greatly enhanced under the influence of anæsthesia. A few drops of blood getting in the trachea would hardly be in ordinary operations of noticeable moment, although in anæsthesia might add seriously to impending evils; but a tooth, or a fragment of a tooth, might readily prove fatal.

There is indeed too many contingencies attending anæsthesia; too little of positive knowledge of the nature and

*modus operandi* of the agents used; too much guess work in the selection of the subjects adjudged fit to undergo anæsthesia, and indeed too slight amount of pain attending extraction of teeth in a normal condition of physic, to justify an encouragement in a common use of general anæsthesia in dental practice. And to discourage an unnecessary and reckless practice, I submit these considerations of the many more that might be adduced.—*Dental Luminary.*

